# A Smart Real-Time Parking Control and Monitoring System

**DOI:** 10.3390/s23249741

**Published:** 2023-12-10

**Authors:** Abdelrahman Osman Elfaki, Wassim Messoudi, Anas Bushnag, Shakour Abuzneid, Tareq Alhmiedat

**Affiliations:** 1 Faculty of Computers & Information Technology, University of Tabuk, Tabuk 47512, Saudi Arabia; w.messoudi@ut.edu.sa (W.M.); abushnag@ut.edu.sa (A.B.); 2 Department of Cybersecurity and Network, School of Criminal Justice Studies, Roger Williams University, Bristol, RI 02809, USA; sabuzneid@rwu.edu; 3 Artificial Intelligence and Sensing Technologies (AIST) Center, University of Tabuk, Tabuk 47512, Saudi Arabia

**Keywords:** smart parking, Internet of Things (IoT), Artificial Intelligence (AI), Optical Character Recognition (OCR)

## Abstract

Smart parking is an artificial intelligence-based solution to solve the challenges of inefficient utilization of parking slots, wasting time, congestion producing high CO_2_ emission levels, inflexible payment methods, and protecting parked vehicles from theft and vandalism. Nothing is worse than parking congestion caused by drivers looking for open spaces. This is common in large parking lots, underground garages, and multi-story car parks, where visibility is limited and signage can be confusing or difficult to read, so drivers have no idea where available parking spaces are. In this paper, a smart real-time parking management system has been introduced. The developed system can deal with the aforementioned challenges by providing dynamic allocation for parking slots while taking into consideration the overall parking situation, providing a mechanism for booking a specific parking slot by using our Artificial Intelligence (AI)-based application, and providing a mechanism to ensure that the car is parked in its correct place. For the sake of providing cost flexibility, we have provided two technical solutions with cost varying. The first solution is developed based on a motion sensor and the second solution is based on a range-finder sensor. A plate detection and recognition system has been used to detect the vehicle’s license plate by capturing the image using an IoT device. The system will recognize the extracted English alphabet and Hindu-Arabic Numerals. The proposed solution was built and field-tested to prove the applicability of the proposed smart parking solution. We have measured and analyzed keen data such as vehicle plate detection accuracy, vehicle plate recognition accuracy, transmission delay time, and processing delay time.

## 1. Introduction and Motivation

Searching for suitable car parking in modern cities is considered a challenge in terms of wasted time and power consumption [1]. In the modern era, car licenses are daily growing at a very high rate, which means the number of vehicles has expanded, which leads to severe traffic conjunctions, and onerousness to find suitable car parking. In modern cities, car parking slots remain as they are, while the number of vehicles is expanding. Hence, modern cities are suffering from car parking shortages. According to [1], in the USA, over 70 billion dollars are wasted every year searching for parking, which is equal to 3.6 billion hours of time and 1.7 billion gallons of fuel. According to [2], one hour of congestion could emission enhancement fluxes of air pollutants and carbon dioxide by 14.3~30.4%.

According to [3,4], the main reason for traffic conjunction in big cities is the car parking problem. Finding a suitable parking space is a challenge in big cities. Therefore, governments should implement laws to address this issue [5]. The developed systems [6,7] have proved the significance of using intelligent parking modes in modern cities. Although modern cities have applied different intelligent solutions for solving car parking issues, the problem still does exist [8].

There are two types of parking spaces: those reserved for specific individuals and free parking spaces available to everyone. Parking spaces reserved for specific individuals may be unoccupied if the person is on leave or absent from work. The problem becomes apparent when the number of employees in an organization is greater than the number of parking spaces allocated to the organization. On the other hand, when the number of visitors exceeds the available parking spaces, visitors may park incorrectly, causing traffic congestion.

The daily parking problems can be summarized as (1) shortage of designated parking slots for certain buildings, which is not enough for employees and visitors. (2) traffic congestion caused by visitors searching for parking slots. (3) Incorrect parking where drivers park vehicles in a spot that is not designated for them. This could happen in a variety of settings, such as in a parking lot where each spot is assigned to a specific individual or in a public street where there are specific regulations about where and how long one can park. Hence, the optimal solution that could deal with the above challenges should: (1) provide dynamic parking slot distributions to address the shortage of parking slots. (2) provide a mechanism for pre-booking by specifying a specific parking slot at a specific time to address the problem of incorrect parking. (3) provide a mechanism to make sure that the car is parked in its correct parking slot, this solution is to address the issue of incorrect parking. In addition, the optimal solution should keep records of traffic patterns in the parking. These steps would formulate a control and monitoring mechanism for a smart parking system. To ensure the success of this mechanism, it must operate in real time.

This paper discusses the design and development of a smart real-time parking slot management system, where the parking slots for an organization’s employees and visitors are defined by an intelligent mobile application. Allocating parking spaces for employees is carried out dynamically, as parking spaces for employees are determined according to work shifts where the staff receives their parking slots through the application. On the other hand, visitors need to request an appointment to be allocated a parking slot. When the car arrives at its predefined parking slot, the parking slot’s camera captures a picture of a car plate. It then sends it associated with time/data to an online database and creates a record. The plate picture is detected and recognized using the intelligent system to get the plate number. The proposed smart parking system provides a real-time database of the parking slot positions in the organization, which enhances parking security. The database will be updated automatically and provide analytical reports for usage. Analytical reports assist in knowing parking patterns, which facilitates the process of creating parking spaces for similar organizations. In this paper, Internet of Things (IoT) Cameras, sensors, and machine learning software (including OpenCV version 4.5.5, and TensorFlow version 2.14.0) have been used to monitor, and control parking.

This proposed dynamic smart parking system could provide different facilities such as: (1) optimizing the parking system by providing dynamic distributions based on real need. (2) could be used to allow/disallow cars in and out while recording the arrival and departure times and obtain a weekly or monthly report for each customer. (3) Cars that do not leave their parking slots can be instantly identified and reported. (4) car localization by the customers. The admin can also inquire about the location of any car at any time. (5) Scheduling car parking slots for visitors. (6) Arrange additional car slot parking from the neighborhood in case of demand.

This paper is organized as follows: Related work has been analyzed and discussed in Section 2. The design of our proposed system has been presented in Section 3. Section 4 contains a description of the proposed system. The conclusion has been presented in Section 6.

## 2. Related Work

The significance of smart parking, both from a theoretical research perspective and from an applied commercial perspective, is reflected in the large volume of research work in this field. Thus, only recent works have been considered, and the analysis of related works is limited to a timeframe of five years, from 2018 to 2023.

In this section, the related works have been analyzed for extracting strengths and weaknesses aiming for highlighting the research gap and providing beneficial directions for researchers interested in this subject. As an exclusion criterion, all works related to smart and electric vehicles have been neglected. These works should consider power consumption and recharging, which are outside the scope of this paper. In addition, works with repeated ideas have been excluded.

The work presented in [9] involves a development of a system based on employing an Artificial Neural Network (ANN) for selecting a suitable parking slot in a real-time environment. The system analyzed employees’ behavior and accordingly suggested a suitable parking slot time and location. This solution lacks a real time control system. The work in [10] developed a system based on drone-based surveillance to find suitable parking slots. This work has a high cost of implementation. The work presented in [11] proposed intelligent parking system by using Arduino sensors. This system is working by connecting chips with the Ethernet W5100 network and then transmits the collected information to the server. This system does not keep records of the movement of cars within the parking lot. The work presented in [12] have developed an intelligent system to find free parking slots. This system is developed based on a detector that consists of three parts: the STM32 microcontroller, the geomagnetic sensor, and the NB wireless module. The geomagnetic sensor collects the intensity of the magnetic field around, and then the magnetic field intensity is sent to the microcontroller. The microcontroller determines whether the parking space is free or not. This solution only includes a service for finding free parking slots. In [13], authors improved Radio Frequency Identification (RFID) by using a low-profile vertical polarized antenna to enhance intelligent parking based on (RFID). This solution is just to assist in finding free parking slots. The work presented in [14] includes the development of an intelligent method based on genetic algorithms for optimizing the selection process for selecting free public parking. The lack of a tracking mechanism could be a significant limitation of this proposal. The work presented in [15] have developed an intelligent parking system based on ultra-wideband (UWB) position and navigation technology. This system is suffering limitations due to the limitation of UWB.

In [16], authors developed an intelligent method for assisting drivers by predicting suitable paths and parking slots. This method has been developed by using the Internet of Things (IoT), and genetic algorithms. It was enhanced by using Artificial Neural Network (ANN). The work presented in [17] have developed a simulation of the shared parking operation that considers the uncertainties with four parameters which are users, arrival, and departure time. The presented work in [18] involves the employment of a canny edge detection method and a USB camera to define the available parking slots. The work in [19] is used wireless vehicle detectors and magnetic sensors to define the availability of parking spaces. The four aforementioned works lack tracking and controlling mechanisms.

The work presented in [20] employed a Raspberry Pi 4 B+ (RPi) computer, Pi camera module, GPS sensor, ultrasonic sensors, and Blynk App to develop an intelligent parking system. This solution is successfully finding the available parking slots over the Internet. The work in [21] has developed an intelligent system that allows users to pre-reservation free parking slots and then update the parking status after detecting a car’s plate by using the OCR algorithm. The work in [22] has developed an intelligent mobile application that can suggest free parking slots around the target location. The suggestions are generated based on the user’s preferences.

In [23], authors have developed a parking management system based on ARM and ZigBee wireless sensor networks to define free parking slots. The deep learning model has been used as a car’s plate recognition system, where the system controls the entrance and exits of the parking. The work in [24] has developed an intelligent model to recognize the free parking slots based on AI vision and the MobileNet classification model. This system can recognize occupied parking slots and the vehicle’s type. The work in [25] has developed an intelligent parking system by integrating related technologies such as ZigBee, geomagnetic sensors, and RNN to detect the status of parking spaces.

The work presented in [26] includes the development of an intelligent model that utilizes a Convolutional Neural Network–Long Short-Term Memory (CNN-LSTM) architecture, along with comprehensive 360-degree panoramic images, ultrasonic and sensor distance measurements. The model is designed to assist drivers in accurately parking their cars, thereby reducing congestion in open parking spaces. In [27], authors have developed an automatic parking lot occupancy detection model. This model utilizes an around-view monitor (AVM) image sequence with a 360-degree bird’s-eye view camera, as well as ultrasonic sensors, to accurately determine the presence of adjacent vehicles.

The optimal solution should provide (1) dynamic parking slot distribution, (2) a mechanism for pre-booking a parking slot, and (3) a mechanism to ensure that the car is parked in the correct slot. (4) inquire about the location of any car at any time. The first two represent control of the parking system, while the third, and fourth pertain to monitoring. Based on the discussion and analysis of related works, it is evident that there is a critical need for a real-time system that provides both control and monitoring subsystems. Therefore, we will use the features of optimal solution as benchmark to compare the proposed smart parking with the related works. Table 1 shows this comparative summary.

## 3. System Design

**Figure 1:**
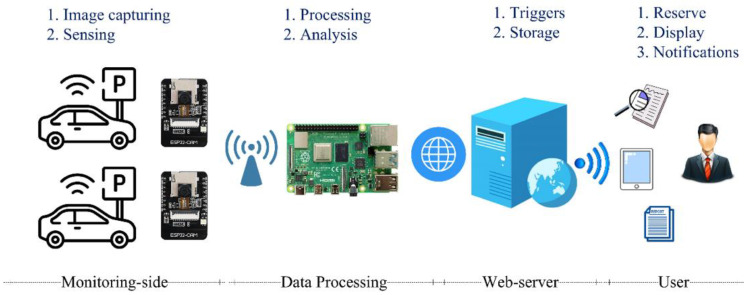
The main architecture of the proposed system.

In this section, a discussion of the design of the proposed smart real time parking controlling and monitoring system has been presented. Figure 1 shows the system architecture, which consists of four main components: the monitoring unit, processing unit, cloud-side unit, and user application. Each component is discussed in the following.

**Monitoring Unit:** The monitoring unit is hosted at each parking slot and consists of a smart IoT device. This low-cost, small-sized, and low-power consumption unit can detect the presence of a vehicle, take a picture, and transmit it to the processing unit.

**Processing Unit:** This unit consists of a low-cost computer (Raspberry Pi 4) that can receive the images taken by the monitoring units, process and retrieve the required information from the images using an Automatic Number Plate Recognition (ANPR) system and transfer the retrieved data to the cloud-side unit to make it available for the user application.

**Cloud-side Unit:** This unit collects the extracted information from the images received from the IoT device, records the final details in the database, and performs the user alerting function. The information includes the vehicle’s license plate number, parking time, and parking slot identity number.

**User Application:** This application has been designed to enable users to perform various functions, such as registration, booking, and modifying bookings.

**Figure 2:**
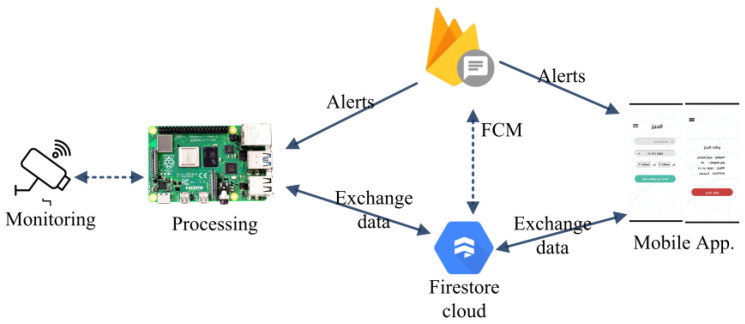
Data exchange between the proposed systems’ components.

Figure 2 depicts the data communication of the proposed system. The monitoring unit captures a photo of the parked vehicle and transmits it to the processing unit (Raspberry Pi 4). The processing unit identifies the vehicle’s license plate and recognizes the characters using Optical Character Recognition (OCR) from PyTorch. Once the characters are identified, a new record is created in the Firestore cloud database, and an alert is sent to the user using Firebase Cloud Messaging (FCM).

## 4. Description of System Operations

The developed smart real-time parking control and monitoring system works as follows: when a car parks in a parking slot, the monitoring unit detects its presence using either the range-finder sensor or the motion sensor. The monitoring unit then captures an image of the parked vehicle and transmits it directly to the processing unit. The processing unit checks the quality of the image and requests a replacement if the received image is of low quality. Afterward, the processing unit processes and analyzes the content of the image to recognize the license plate number.

After recognizing the license plate number, the car’s details, such as the license plate number, parking slot number, time, and date, are transmitted to the cloud server (Firebase in our case) to keep a record of the parked vehicle. The Firebase cloud server then triggers a notification to check the driver’s record to ensure that they have parked in the correct slot. If the driver has parked in the wrong slot, a notification message will be sent to both the driver’s mobile application account and the administrator. On the other hand, when a visitor requests a parking slot, the mobile application sends a request to the Firebase cloud server, which approves the request.

### 4.1. Hardware Design

The developed parking system involves providing two different parking devices that have been developed using the same microcontroller. However, a slight difference has been made in the employed sensing technology to sense the presence of a vehicle. In other words, the proposed system is available in two different modules, aiming to provide cost flexibility.

**Figure 3:**
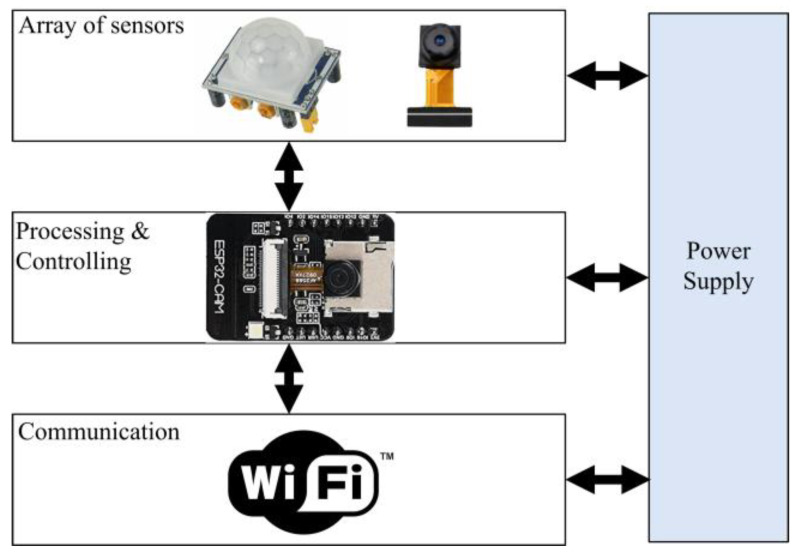
First module architecture with motion sensor.

First Module: Using motion sensor. The first module architecture is presented in Figure 3, which uses a motion sensor, and ESP32-CAM-M5-Stack board. The motion sensor is efficient in terms of detecting the presence of a vehicle, however, the motion sensor fails to identify the object’s identity and cannot find the distance to the car plate. However, this module provides reduced cost in comparison with the second module.

**Figure 4:**
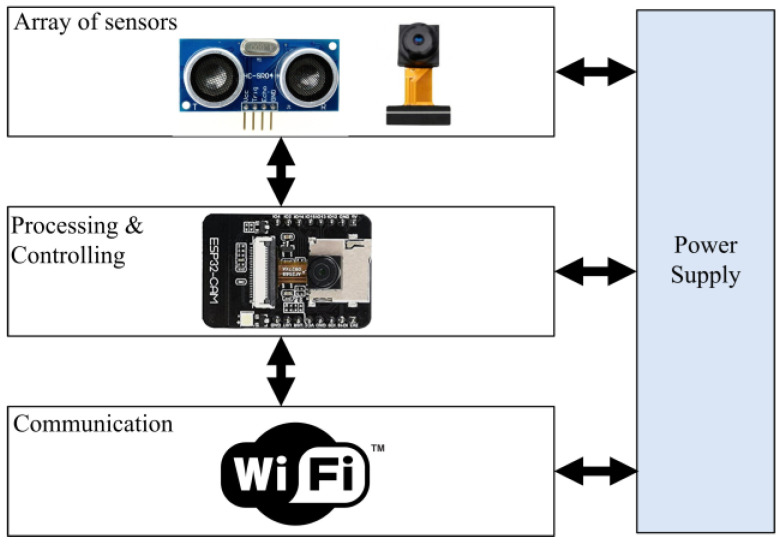
Second module architecture with range-finder sensor.

Second Module: Using range-finder sensor. The second module architecture is presented in Figure 4, which consists of an ESP32-CAM-M5-Stack board and a range-finder sensor. The range-finder sensor detects the presence of a vehicle in a parking slot, and the ESP32-CAM module will take a photo of the car’s license plate.

**Figure 5:**
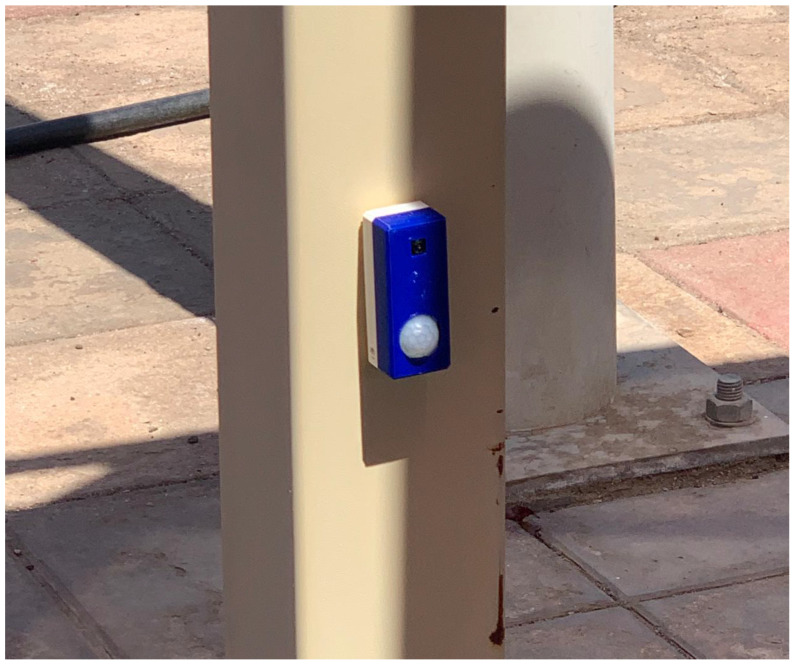
Monitoring using motion sensor.

**Figure 6:**
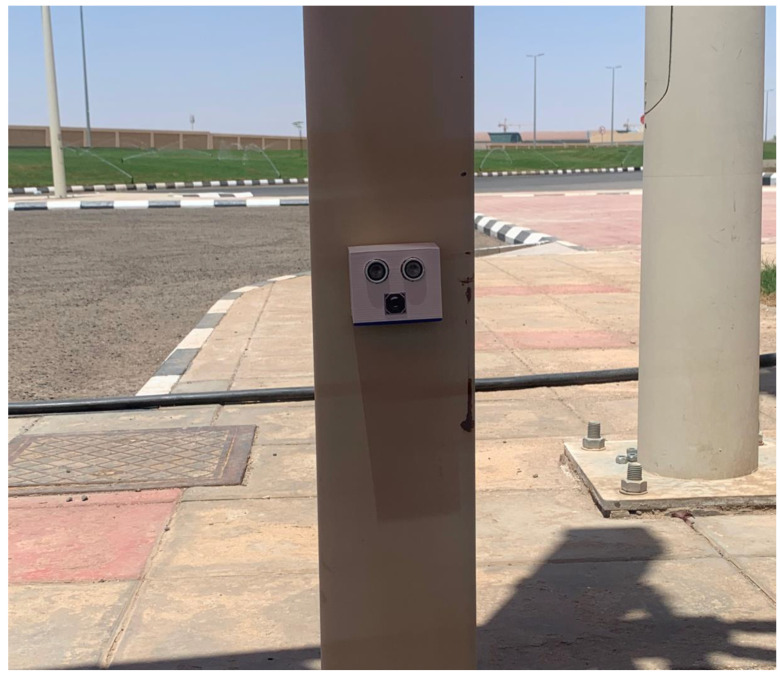
Monitoring using range-finder sensor.

The main difference between the two units lies in their sensing capabilities, specifically the method employed to detect the presence of vehicles. The developed monitoring in the first module is depicted in Figure 5, while Figure 6 showcases the developed monitoring in the second module.

For implementation purposes, and as shown in Figure 3 and Figure 4, the code for the smart parking system was divided into three main parts:

**Part 1:** The IoT code that runs on the ESP32-CAM module involves the commands for collecting the signals from onboard sensors (range-finder or motion), in addition to the code that takes a picture with a certain resolution (1024 × 768) pixel, and the piece of code that transmits the taken image to the Firebase server. Table 2 presents the Pseudo code for the Passive Infrared sensor (PIR-based) monitoring unit, whereas Table 3 shows the Pseudo code for the Range-based (R-based) monitoring unit.

**Part 2:** Firebase code: this involves a set of commands to establish communication between the IoT device and the Firebase cloud storage. Table 4 presents the pseudocode for the upload function.

**Part 3:** Computer vision code: This involves a set of commands to detect the car’s license plate, preprocess the image, and recognize the characters. More details will be presented in Section 4.3.

### 4.2. User Application

**Figure 7:**
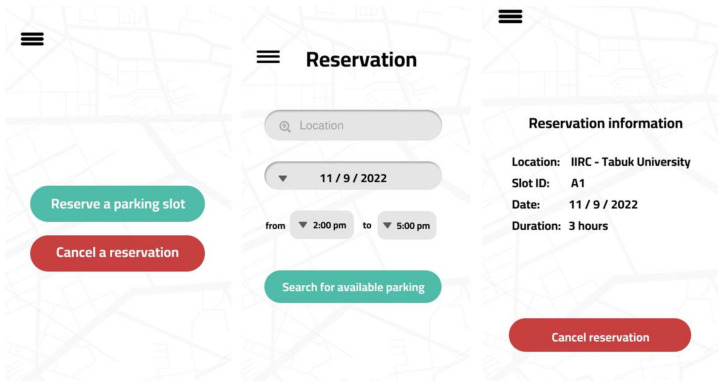
Screenshot of the User Application.

The user application module allows users to interact with the developed smart parking system. The user application has been developed using a cross-platform environment (Flutter) to facilitate communication with the users efficiently. Figure 7 presents a screenshot of the developed application that is required to accomplish the parking reservation process.

**Figure 8:**
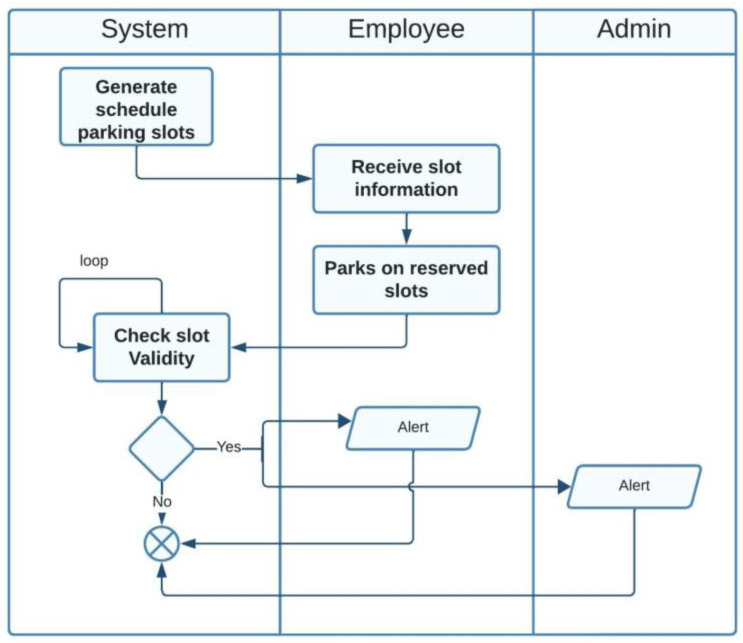
Activity diagram for the employee actor.

**Figure 9:**
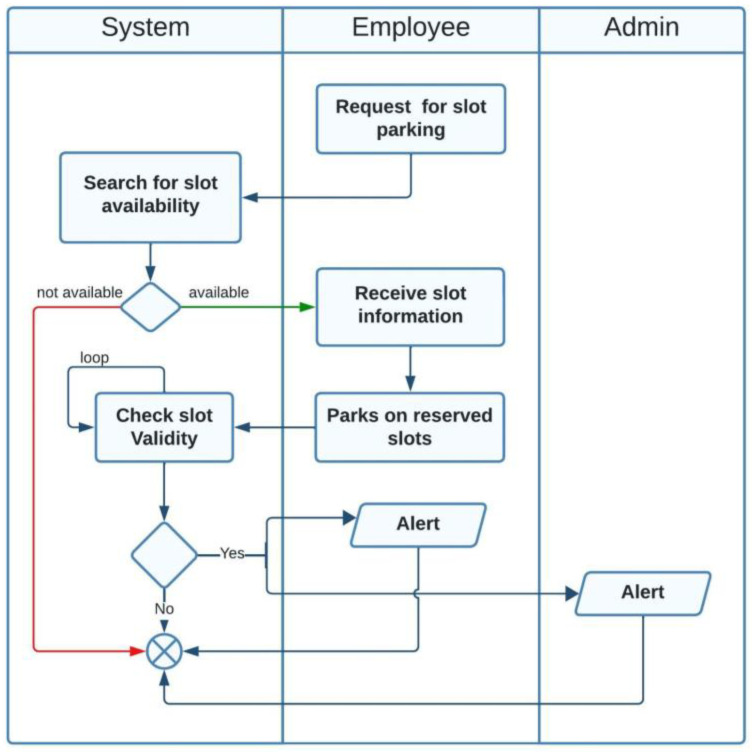
Activity diagram for the visitor actor.

The actors in our case are employees, visitors, and administrators. Two possible scenarios are available for parking slot reservations as illustrated in Figure 8 and Figure 9.

In the first scenario, the system distributes slot information to employees according to their official work hours or shift hours, using a priority queue data structure. Then, the employee parks in the assigned slot. The system checks if the employee has occupied the correct slot; if not, an alert will be sent to both the employee and the administrator.

The second scenario is related to the visitor actor. First, the visitor requests a parking slot. Then, the system checks the availability and sends the slot information to the visitor.

### 4.3. Plate Detection and Recognition System

For the plate detection task, we developed an Automatic Number Plate Recognition (ANPR) system that consists of three main functions.

▪ Detect and localize the vehicle’s license plate in the received image from the IoT unit.
▪ Extract the characters (numbers and letters) from the detected image.
▪ Apply the Optical Character Recognition (OCR) model to recognize the extracted characters.

**Figure 10:**
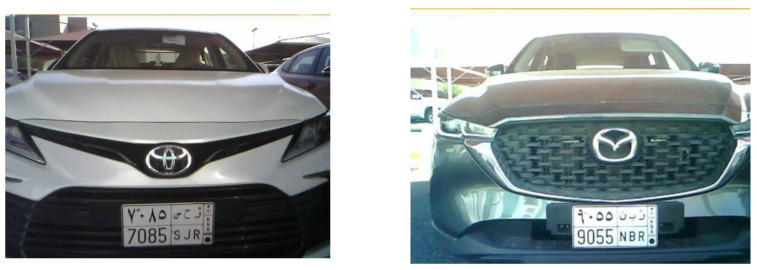
Examples of two vehicle plates detected using the monitoring unit.

The detection and localization of the vehicle’s license plate have been accomplished using the SSD MobileNet V2 detection model. This model is a single-stage object detection model that has been widely developed for low-compute capability devices such as smartphones and small computers, offering high accuracy performance. In our case, we employed the SSD MobileNet V2 detection model on Raspberry Pi 4 to detect the vehicle’s license plate in the captured images obtained from the monitoring unit. Figure 10 displays two images of two different vehicles, collected from the monitoring unit.

**Figure 11:**
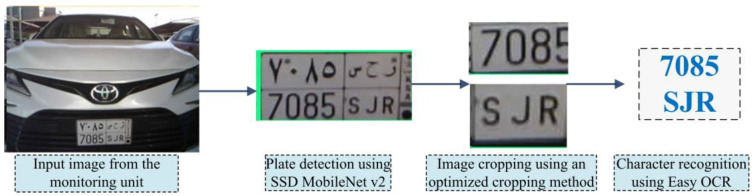
The main stages of the developed ANPR system.

To achieve high accuracy and minimize the processing time for plate detection, we trained the SSD MobileNet V2 model on the car license plate detection dataset [28]. This dataset consists of 433 images with bounding box annotation of the car lenience plates within the image. Therefore, the training process was performed using an input of 433 different car images. Figure 11 shows the main stages of the developed ANPR system.

For character identification, we employed the Easy Optical Character Recognition (EasyOCR) engine to recognize English letters and Arabic numbers. EasyOCR is a Python-based library developed for OCR tasks, and it is capable of accepting the license plate picture and performing optical character recognition on it immediately.

**Figure 12:**
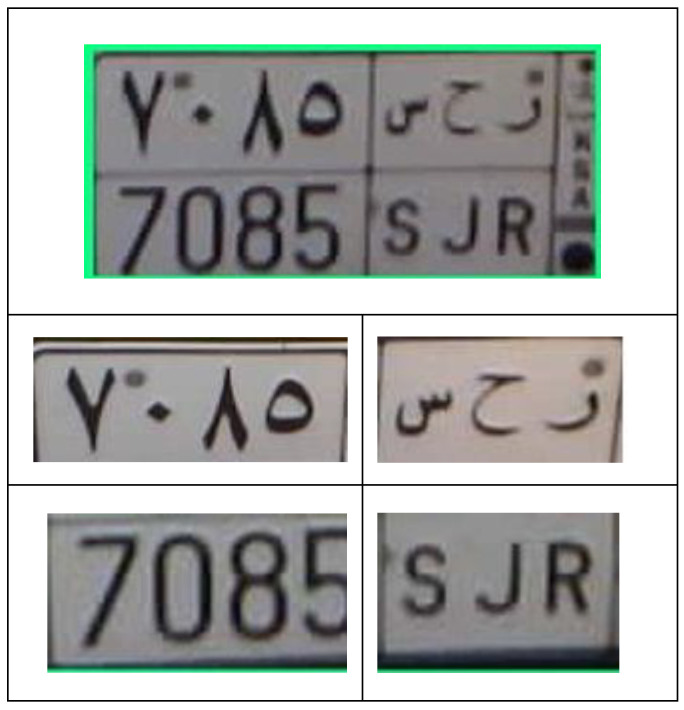
The process of cropping a vehicle’s plate.

In the first stage, we obtain the detected license plate from the previous stage (plate detection function) and then feed it into the Easy OCR engine. However, the obtained results were not accurate in terms of recognition accuracy. Therefore, we implemented a crop function to divide the detected plate into four different sections: English alphabet and Hindu-Arabic Numerals. These sections are illustrated in Figure 12. Table 5 presents the Pseudocode for the developed ANPR system.

The above functions are processed in the processing unit (Raspberry Pi computer in our case). Then, the processed data are transferred to the cloud-side unit in order to further process the received data and allow them available for user-side.

## 5. Experiments

This section discusses both the experiment testbed and the results obtained from various experiments conducted in a real parking area belonging to the Faculty of Computers and Information Technology located on the main campus of the University of Tabuk. As mentioned earlier, we designed and employed two different monitoring units. These experiments have been conducted using the second module. Table 6 displays the main parameters setup for the real experiments conducted in several parking areas.

### Experimental Results

This section discusses the results obtained from various experiments conducted to evaluate the accuracy of plate recognition. For experimental analysis results, we tested the following parameters:

1. Vehicle plate detection accuracy: This refers to the number of plates that were correctly detected out of the total number of vehicle plates.
2. Vehicle plate recognition accuracy: This refers to how effectively the recognition system was able to identify and recognize the characters and numbers on the plate.
3. Transmission delay time: This refers to the average time required to capture the vehicle’s plate and transmit the captured image to the processing unit.
4. Processing delay time: This refers to the average time required to receive and process the captured image by the IoT monitoring unit.

**Figure 13:**
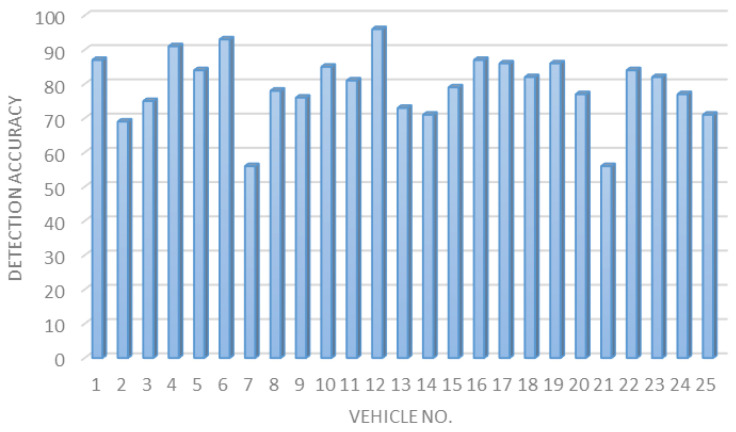
Plate detection accuracy for 25 different vehicles.

**Vehicle plate detection accuracy.** The obtained results for 25 different car plates for vehicle plate detection are presented in Figure 13.

**Figure 14:**
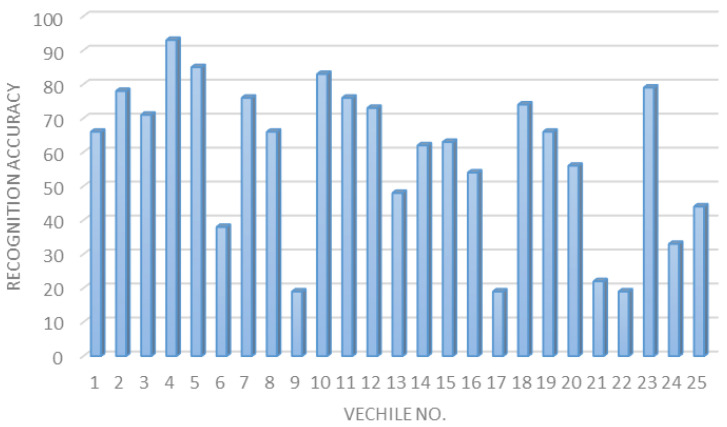
Plate regression accuracy for 25 different vehicles.

**The vehicle plate recognition accuracy.** As discussed earlier, the EasyOCR has been employed in our experimental testbed. However, according to the conducted experiments, the EasyOCR offers limited detection accuracy, as shown in Figure 14. The estimated average plate recognition accuracy was around 58.52% which is a low value and may offer inefficient recognition accuracy.

**Figure 15:**
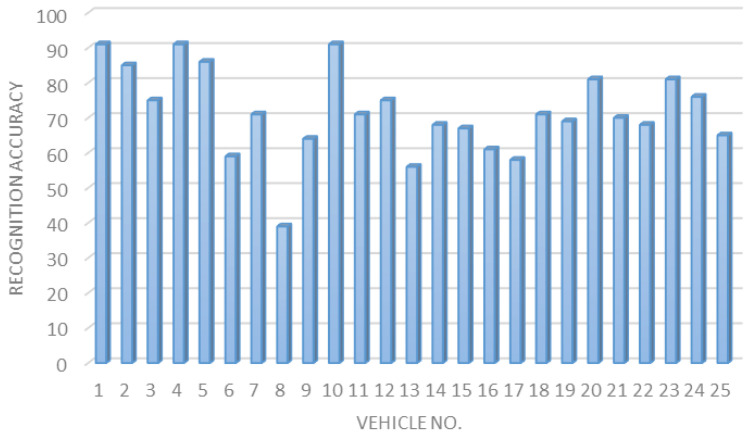
Enhanced plate regression accuracy for 25 vehicles.

To improve the regression accuracy of the proposed smart parking system, we implemented a plate division function that splits the vehicle’s plate into two sections: English alphabet and Hindu-Arabic Numerals. Figure 15 illustrates the plate regression accuracy for 25 different vehicles, showing a significant enhancement of 14% in the regression accuracy.

**Figure 16:**
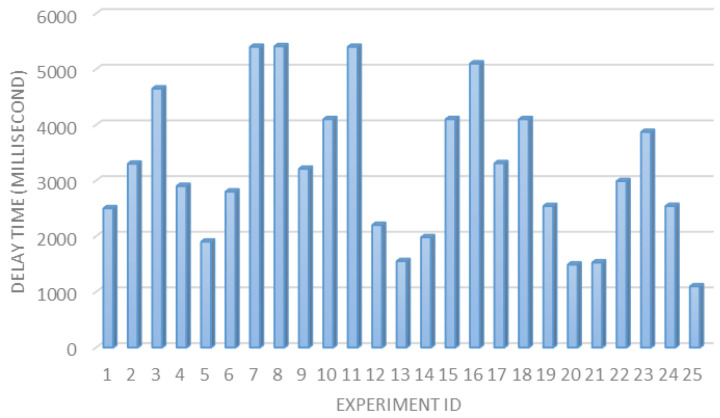
Delay time in milliseconds for 25 experiments.

The transmission delay time. In general, it is important to study and analyze the time required to capture and transfer the vehicle’s plate image to the processing unit (Raspberry Pi computer). Figure 16 presents the delay time for 25 trails in different parking areas with an average delay time of 3198 milliseconds (almost 3 s).

**Figure 17:**
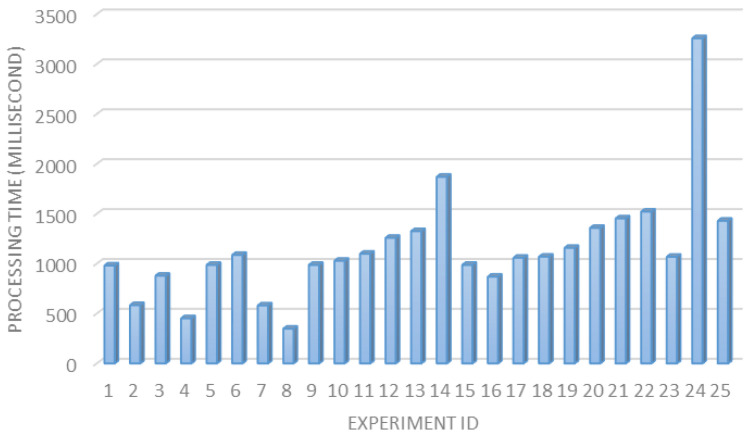
Processing time (including plate identification and recognition) for 25 experiments.

The processing delay time. The captured images need to be processed and analyzed to extract the characters and numbers from the license plates. Therefore, it is important to study and analyze the average processing time required for this task. Figure 17 displays the processing time for 25 trials conducted in the experiment testbed, with an average processing time of 1147 milliseconds and an average delay time of 1 s.

Table 7 presents the main specifications for monitoring units 1 and 2. As presented in the Table below, the main difference between the two units involves the adopted sensing method, the ability to measure the distance to the vehicle, and the cost. For the range-finder system, the success rate is almost 89%, whereas the success rate for the motion-based system is almost 51%.

## 6. Conclusions and Discussion

According to [7], the fusion of sensor-based parking slots, plate recognition, and range sensors is expected to be the most important approach in the near future. In this paper, a smart real-time parking control and monitoring system has been introduced.

This proposed system is smart from three perspectives: First, the distribution of car park slots is based on real-time demand. The distribution process is dynamic and takes into account employees’ working shifts, optimizing car slot usage and addressing the issue of parking slot shortage. Second, provide pre-booking for a parking slot. Third, the system provides automatic parking lot occupancy detection, which enables verification of whether a car is parked in its correct parking slot or not. By incorporating the dynamic distribution of parking slots, prebooking, and automatic parking lot occupancy detection, the proposed system enhances the overall efficiency, security, and management of the parking facility, providing a seamless parking experience for users while ensuring proper utilization of parking spaces.

The contribution of this proposed smart could be summarized as follows: providing a low-cost IoT monitoring unit has been implemented using a low-cost microcontroller. Providing minimum delay in the data exchange and processing tasks. Providing efficient recognition accuracy has been achieved. In the following, our contribution will be highlighted by using benchmark consist of four parameters which real time monitoring, real-time controlling, speed, and cost. Below, definition of each parameter has been presented.

- Real-time Monitoring: Monitoring a vehicle when it is parked at parking slot and when it moves out, at real time.
- Real-time Controlling: Controlling parking by preventing incorrect parking.
- Speed: What is the speed of capturing, processing, and recognition functions?
- Cost: What is the cost of the hardware unit per slot.

In despite of the related works, our proposed smart parking system has satisfied these four criteria. Discussion is as follows:

Real-time Monitoring: smart IoT device. This low-cost, small-sized, and low-power consumption unit can detect 184 the presence of a vehicle, take a picture, and transmit it to the processing unit.

Real-time Controlling: Controlling parking by sending messages (from our developed application) to the driver when he parks in the wrong parking slot, or when his time is over. Hence, the integration of mobile application with developed hardware model creates the controlling unit in our proposed smart parking system.

Speed: Our proposed system is very fast due to two reasons: Firstly, the monitoring unit is hosted at each parking slot. Secondly, using a cloud-side to collect the extracted information and performs the user functions.

Cost: According to Table 7, the cost of our proposed system is 12.00 USD per slot.

Clearly, Table 1 and Table 8 reflect and substantiate our contributions.

As mentioned earlier, our proposed system can be implemented in two different modules. The first module utilizes motion sensors for monitoring, while the second module utilizes range-finder sensors. The main difference between the two modules is the cost, with the first module being cheaper by two US dollars per slot. The range-finder-based system is much better than the Motion-based system for the following reasons:

1. The motion-based system is very sensitive to any movement that may act heading the monitoring unit.
2. Range-finder system can detect the distance to the vehicle’s plate and therefore a high-precision image is guaranteed.

The success rate for the motion-based system is less than for the range-finder system. This is because the motion sensor can be affected by any object heading the motion sensor. However, the ranger-finder sensor achieves a better success rate, as the ranger-finder sensor estimates only the approaching vehicles to the monitoring unit, and this minimizes the false acceptance rate.

As aforementioned, in our proposed smart parking system, the parking space is dynamic and flexible. For instance, consider three organizations A, B, and C, each having their own parking space. Now, suppose there is a special celebration where there is a critical need for a larger parking space. In our application, we can define these three individual parking spaces as a combined parking space. This concept can also be implemented in public parking areas.

As part of our future work, we are currently focusing on implementing smart parking solutions for electric vehicles (EVs). This involves addressing two new challenges specific to EVs: the need for special car park slots equipped with energy producer sources and accommodating the charging time requirements of each EV.

## Figures and Tables

**Table 1:** Comparative summary between related works and the proposed system.

The Work	Dynamic Distribution	Pre-Booking	Ensure the Correct Slot	Inquire about the Location of Any Car at Any Time
[9]	X	√	X	X
[10]	X	√	X	X
[11]	X	X	√	X
[12]	X	√	√	X
[13]	X	√	X	X
[14]	X	√	√	X
[15]	X	X	√	X
[16]	X	√	√	X
[17]	X	√	X	√
[18]	X	√	√	X
[19]	X	√	√	X
[20]	X	√	X	X
[21]	X	√	√	X
[22]	X	√	√	X
[23]	X	√	√	X
[24]	X	X	√	X
[25]	X	X	√	X
[26]	X	√	X	X
[27]	X	√	X	X
The proposed system	√	√	√	√

**Table 2:** Pseudocode for PIR-based monitoring unit function.

define imagePath Function main_loop() { while(true) motionDetected = get_PIR_sensor_status() if(motionDetected == true) capture_image() upload_image_to_firebase_cloud_storage() }

**Table 3:** Pseudocode for the R-based monitoring unit.

define imagePath Function main_loop() { while(true) distance = get_dist_to_object() if(distance<=100) capture_image() upload_image_to_firebase_cloud_storage() }

**Table 4:** Pseudocode for the upload function.

define imagePath Function upload_image_to_firebase_cloud_storage() { capture_image_and_save_to_file_system() Firebase_storage_upload(imagePath) }

**Table 5:** Pseudo code for the developed ANPR system.

Function main () { define imagePath plateImage = detect_plate(imagePath) extractedNums = get_numbers_from_plate_image(plateImage) extractedChars = get_chars_from_plate_image(plateImage) } Function detect_plate(imagePath) { detecBoxCoords = plate_detection_model(imagePath) plateImage = crop_plate_part(detectionBoxCoords) return plateImage } Function get_numbers_from_plate_image(plateImage) { plateLowerPart = get_plate_lower_half(plateImage) plateNumsPart = get_plate_left_half(plateLowerPart) allowedChars = [0,1,2,3,4,5,6,7,8,9] extractedNums = extract_using_EasyOCR(plateNumsPart, allowedChars) return extractedNumbers } Function get_letters_part_from_plate_image(plate) { plage_LowerPart = get_plate_lower_half(plateImage) plateLettersPart = get_plate_right_half(plateLowerPart) allowedChars = [A,B,C,D,E,F,G,H,I,J,K,L,M,N,O,P,Q,R,S,T,U,V,W,X,Y,Z,a,b,c,d,e,f, g,h,i,j,k,l,m,n,o,p,q,r,s,t,u,v,w,x,y,z] extractedLetters = extract_using_EasyOCR(plateLettersPart, allowedChars) return extractedLetters }

**Table 6:** The main experiment parameters.

Specification	Value
IoT module	ESP32-CAM
Camera resolution	2 MP (1600 × 1200)
Image resolution	1024 × 768
Communication protocol	IEEE 802.11

**Table 7:** The main specifications for monitoring in the two developed modules.

	Module 1 (Range-Finder-Based)	Module 2 (Motion-Based)
Microcontroller	ESP-32	
Camera-sensor	4 MP	
Sensing method	Ultrasonic (HC-SR04)	PIR motion sensor
Distance to vehicle	√	×
Cost	USD 13.00	USD 12.00

**Table 8:** A comparison between the developed system in this paper and the recent developed systems.

Related Work	Real-Time Monitoring	Real-Time Controlling	Speed	Cost
[9]	Not defined	Not defined	Processing in central computer sever	Not defined
[10]	Not defined	Not defined	Acceptable	Use drone-based surveillance. (High cost)
[11]	Not defined	Not defined	Processing in central computer sever	Use connecting chips with the Ethernet W5100 network. High cost.
[12]	STM32 microcontroller	Not defined	Processing in central computer sever	Use STM32 microcontroller, the geomagnetic sensor, and the NB wire-119 less module. High cost
[13]	Use RFID	Not defined	Processing in central computer sever	Use low-profile vertical polarized antenna and RFID(High cost)
[15]	UWB	Not defined	Not defined	Use ultra-wideband (UWB) position and navigation technology(High cost)
[16]	RFID	Not defined	Not defined	Not defined
[17]	IOT devices	Not defined	Processing in central computer sever	Not defined
[18]	USB camera	Not defined	Not defined	Not defined
[19]	wireless vehicle detectors	Not defined	Computer sever	wireless vehicle detectors and magnetic sensors
[20]	Pi camera module	Not defined	Raspberry Pi	Raspberry Pi 4 B+ (RPi), Pi camera module, GPS sensor, ultrasonic sensors
[21]	Pi Camera	Not defined	Not defined	Not defined
[22]	IOT devices	Not defined	Computer server	Not defined
[23]	Wireless sensors	Not defined	Computer server	ARM and wireless sensor networks
[24]	IOT sensors	Not defined	Not defined	Wireless sensor networks and camera
[25]	IOT sensors	Not defined	Not defined	geomagnetic sensors and Wireless sensor networks
[26]	IOT devices	Not defined	Not defined	3D camera, ultrasonic and sensor distance measurements
[27]	IOT devices	Not defined	Not defined	3D camera, ultrasonic and sensor distance measurements
